# Pedestrian Detection with Multi-View Convolution Fusion Algorithm

**DOI:** 10.3390/e24020165

**Published:** 2022-01-22

**Authors:** Yuhong Liu, Chunyan Han, Lin Zhang, Xin Gao

**Affiliations:** 1 School of Software, Northeastern University, Shenyang 110167, China; 1971138@stu.neu.edu.cn (Y.L.); 2071346@stu.neu.edu.cn (L.Z.); 2 School of Mechatronics and Information Engineering, China University of Mining and Technology, Beijing 100083, China; skgxin@gmail.com

**Keywords:** autonomous driving, multiview, pedestrian detection, convolution fusion, keypoints

## Abstract

In recent years, the pedestrian detection technology of a single 2D image has been dramatically improved. When the scene becomes very crowded, the detection performance will deteriorate seriously and cannot meet the requirements of autonomous driving perception. With the introduction of the multi-view method, the task of pedestrian detection in crowded or fuzzy scenes has been significantly improved and has become a widely used method in autonomous driving. In this paper, we construct a double-branch feature fusion structure, the first branch adopts a lightweight structure, the second branch further extracts features and gets the feature map obtained from each layer. At the same time, the receptive field is enlarged by expanding convolution. To improve the speed of the model, the keypoint is used instead of the entire object for regression without an NMS post-processing operation. Meanwhile, the whole model can be learned from end to end. Even in the presence of many people, the method can still perform better on accuracy and speed. In the standard of Wildtrack and MultiviewX dataset, the accuracy and running speed both perform better than the state-of-the-art model, which has great practical significance in the autonomous driving field.

## 1. Introduction

Whether for ADAS or autonomous driving, pedestrian detection has traditionally been an unavoidable problem. In the process of vehicle operation, if we can accurately detect the position of each pedestrian, it will greatly guarantee the safety of the autonomous driving field. Therefore, pedestrian detection has become a hot topic in autonomous driving over the past 20 years. The mobility of pedestrians makes them less predictable than vehicles. In the task of pedestrian detection, occlusion has traditionally been the focus of attention. For example, it is difficult to predict the location of all pedestrians under the intersection, since the pedestrians may be blocked by vehicles or other pedestrians. Occlusion is a thorny problem in pedestrian detection, which requires higher accuracy and speed. In the past two years, many people have proposed their solutions on pedestrian detection problems. Existing pedestrian detector algorithms have achieved satisfactory results on standard non-occluded pedestrians, but their performance on heavily occluded pedestrians is far from satisfactory.

To solve the occlusion problem of pedestrian detection, Zhang proposed a two-stage detection framework of Faster R-CNN [1]. Xie used the pedestrian topological structure to solve the pedestrian occlusion problem [2]. These methods perform better than traditional detection models in occluded pedestrian detection. However, they can never break through the limitation of single-camera detection. When the occlusion range is large, the above methods also produce the phenomenon of missing object or false detection. To perform better, the single-camera detection model often contains a lot of redundant calculations, which is defective for salving occlusion problems. There is still much room for improvement in pedestrian detection.

For reasonable control or use, we use the information obtained by multiple cameras in time according to some criteria to obtain the consistent interpretation of the detected target. Because a single camera cannot meet the demand of target detection, if the data collected by different cameras are processed independently, the workload of data processing will increase dramatically. The connection between cameras will be cut off, resulting in the waste of data resources. Therefore, data fusion of multi-camera data is needed to get more reliable conclusions. However, there is no unified standard for the level of data fusion, which is generally divided into data fusion, feature fusion, and result fusion. Among multi-view aggregation, the first proposed method is to fuse the detection results of each camera to obtain the consistent interpretation and description of the target. However, this fusion method is the most inaccurate due to data compression, so it has not been used since it was proposed. The anchor box fusion method proposed later has been greatly improved compared with that before. Nevertheless, the prediction result is not very accurate due to the deformation of the anchor during the fusion, so feature fusion is used later. Feature fusion is used to extract representative features to obtain feature vectors and then fuse these feature vectors without losing important information, which is conductive to real-time processing.

At this point, we turn our attention to the multi-view approach. In theory, the multi-camera detection method can solve the occlusion problem almost perfectly. Multi-view detection contains information of multiple positions, and different cameras provide the target information of each view. Classic models of various perspectives, such as MVCNN [3] and VMVCNN [4], mainly extract features from images of 3D objects from different perspectives and then classify and recognize objects according to the integrated global features. In this way, the model can obtain high-level global features and details. The above method involves the 3D reconstruction method, which uses 3D reconstruction to aggregate information from multiple perspectives. However, the reconstruction process takes a lot of time, and how to use the data from multiple perspectives without taking too much time becomes a significant concern. RCNN and clustering presented a new multi-view and multi-target tracking method, which represented object trajectories as combinatorial hierarchies and probabilistic constraints that characterize the geometry, appearance, and motion of the trajectories [5]. DeepMCD used an architecture that combines multiple instances of it on a small multi-camera dataset [6]. However, none of them well in accuracy. MVDet found that combining a large convolution kernel and the convolution layer with a large receiving field can have a better effect on time and accuracy [7]. However, there is still plenty of room for improvement in accuracy and speed.

As for the analysis above, this paper adopts multi-view target detection to solve the occlusion problem between pedestrians. It adopts the CNN model and large convolution kernel spatial aggregation method and uses feature projection and channel cascade to gather multi-camera information. To better fit the data features, we proposed a double-branch feature fusion model. We replace positive sampling points with keypoints [8], and adopt the Anchor free mechanism to avoid unnecessary accuracy loss caused by anchor frame deformation during multi-camera perspective transformation. In the model, the mid-point of bbox is used to represent the keypoint, which not only solves the occlusion problem but also makes some contributions to the speed of detection.

In addition, we also conduct a series of experiments to prove our conclusion. On the one hand, we explore the respective effect of different factors in the network structure, including the multi-scale convolution fusion and the center points structure. On the other hand, we test the influence from the different datasets. According to the results on the Wildtrack, our final model improved by 1.8% on MODA, 0.5% on MODP, and 0.349 s faster on the test set. According to the results on the MultiviewX, our model improves by 5.6% and 3.8% respectively on MODA and MODP and 0.196 s faster on the test set.

In conclusion, our main contributions are:

(1) A double-branch feature fusion structure is proposed further to extract features for better performance of the model;
(2) The proposed method of center points makes the model detection more flexible and does not need to be limited by the anchor frame mechanism. The method adopts with the multi-scale fusion module, which gives better results when combined;
(3) The model runs faster on the basis of improved model performance, which is more in line with the urgent needs of pedestrian detection.

## 2. Realted Work

With the rapid advancements in deep learning, a plethora of work has been published on pedestrian detection. Such models include region proposal networks such as Faster-RCNN [9], single-shot detectors such as SSD [10] and YOLO [11], as well as pose-estimation models like DeeperCut [12] and OpenPose [13]. Tang proposed a set of methods using gait characteristics to detect pedestrians, and these methods performed well in pedestrian detection [14]. However, it cannot be maintained in the case of occlusion, so some people provide new ideas for the occlusion problem.

As for the pedestrian detection, the occlusion problem is mainly solved by single-view detection. At present, the entry point of the single-view detection method includes dividing the target candidate box into different parts, and then integrating the features. For example, the bi-box [15] method enables the network to output the visible part of the target candidate box, and guide the network to have more discriminating power to the pedestrian target under occlusion in the learning process. Perspective of loss makes the target candidate box more discriminant in order to stay away from each other. Repulsion loss [16], for example, works by setting the loss function forecasting responsible for the distance of the frame of objects, and, together with the surroundings, is not the actual target box (the box that contains natural objects and predict boxes) of space used to improve the model performance. Yang proposed a partially sensing multi-scale fully convolutional network to solve these occlusion and large-scale problems [17]. The most responsive part is selected by voting, and partially visible pedestrian instances can obtain a high detection confidence value, making it unlikely to miss detection. However, the above methods are in a single view, even if the model has a good performance in the case of no serious occlusion problems, it still cannot meet the requirements of autonomous driving for pedestrian detection. In the actual scene, it cannot guarantee that pedestrians are always slightly occluded.

In recent years, multiple perspectives are used to obtain information about the surrounding environment. Similarly, multi-camera targets detection is used to solve the problem of pedestrian occlusion. Chen used a laser point cloud and RGB images as the input to predict the fusion frame of the directional three-dimensional bounding box [18]. Sparse Dd point cloud data is encoded with compact multi-view. Ku used a virtual multi-view synthesis method to generate a set of virtual views for each detected pedestrian [19]. These views are used to create accurate position estimates during training and reasoning. The methods mentioned above are valid on occlusion through multi-view information, but the fusion of laser point clouds and the generation of virtual multi-view took too long. The data collected by real multi-cameras is used to detect pedestrians [7], and the aggregation of multi-perspective information is completed by feature projection. There is still room for improvement in Recall rate and speed. The common point of these methods is that the target of each view is matched in horizontal space, and this matching process is the process of multi-view information fusion. Since the matching operation of the above method occupies a large amount of memory and time resources, its actual use has many limitations.

To aggregate the results obtained from multi-view detection, the multi-view occupancy estimation method is adopted. POM-CNN uses multi-view Bhutan, the Probabilistic Occupancy Map [20], which operates the Bayesian formula to calculate the probability of the existence of objects on the plane. However, the computation speed is plodding and cannot run in real-time. Peng combined a separate Bayesian network to construct an occupancy diagram based on ground position and geometric constraints [21]. Ge used the maximum posterior estimation to find the best-fit occupancy diagram for the image observation [22]. Deep-Occlusion uses the mean-field to infer the complete factorization distribution Q, which approximates a posterior P, to produce a probabilistic occupancy diagram [23]. However, the result of the generated probability occupancy diagram is not intuitive, and it must be processed first to make use of the information of the occupancy diagram.

In our work, we focus on the processing feature and use keypoints to improve the speed and accuracy of the model. A top view and the pedestrian occupancy rate are used to estimate the occupancy diagram to make the result more intuitive.

## 3. Methods

In this work, we focus on the occluded pedestrian detection problem in a multiview scenario. The method aggregation uses results from each camera through projection from the double-branch model structure. Our model takes multiple RGB images as inputs, and outputs the pedestrian occupancy map estimation. In the following sections, we will introduce the model structure (Section 3.1), multi-scale convolution fusion network (Section 3.2), and the center points (Section 3.3).

### 3.1. Model Structure

**Figure 1:**
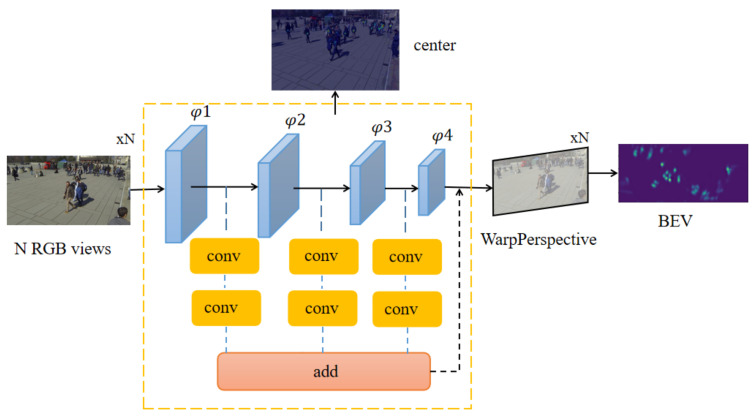
Description of the model structure.

On the basis of maintaining the speed of the original model, our model adopts a double-branch structure to optimize the MODA of ResNet-18 (Figure 1). We apply an anchor-free multiview aggregation that alleviates the influence from inaccurate anchor boxes in the previous work. We propose a double-branch model as the backbone and use the center point instead of anchors to verify the accuracy of our model. In each view, we detect pedestrians with a shared weight single view detector. Finally, through perspective transformation, the concatenate features after the projection are transferred to the same coordinate system to get a bird’s eye view (BEV).

First, given input images of three channels from the *N* cameras, the proposed network uses a double-branch feature fusion structure to extract feature maps for each input image. The CNN feature extractor shares weight among *N* inputs. Next, we run single view detection by detecting the center points. We take an anchor-free approach, and use the center point to regress some characteristic of target boxes. The multiview aggregation approach uses perspective transformation to project N feature maps according to corresponding camera calibrations 1-*N*. Then, we concatenate the *N* projected feature maps and use a convolution to get the result.

The model processes the images of each camera and extracts features through two-layer convolution, respectively, after the double-branch feature fusion network. One layer of convolution gets the center point of regression, the other layer gets the information in the corresponding aerial view of the target. We calculate and add the two results, respectively, to obtain the final loss for reverse propagation, and the final aerial view is obtained after the training.

Our model needs some information, such as camera internal and external parameters and through two convolution extract feature. It then transforms the feature map to a planform. We then compete the object regression loss and the planform location loss to fine tune the final results. The entire process of multiview pedestrian detection is summarized in Algorithm 1.
**Algorithm 1** Multi-view pedestrian detection**Input:** $image_{1}\ldots image_{N}$, Camera internal and external parameters;Parameter C represents the function of extracting features;Parameters L1, L2, and L represent the loss of center points;occupancy map loss and total loss;**Output:** 
res is constant of images from different perspectives1:initial i=0;2:$W_{i}^{c1}$ represents the convolution of 1*1;3:$W_{i}^{c2}$ represents the convolution of 3*3;4:**repeat**5:    compute the $feature_{1}:$$f1=W_{i}^{c1}\left(image_{i}\right)$;6:    compute the ans:$f1=W_{i}^{c2}\left(f1\right)$;7:    add ans to prediction;8:    compute the $feature_{2}:$$f2=W_{i}^{c1}\left(image_{i}\right)$;9:    compute the ans:$f1=W_{i}^{c2}\left(f2\right)$;10:    ans=warpPerspective(f1);11:    add ans to res;12:**until** (i>=N)13:L1=smoothl1(prediction,label);14:compute the perspective loss L2=gussainl(res,label);15:compute the total loss L=L1+L2;

### 3.2. Double Branch Convolution Fusion Network

In this part, the feature fusion method is adopted to preserve the shallow information of the model. According to the existing conclusion, a 3 × 3 convolution has the best result for feature extraction, so we use a 3 × 3 size convolution kernel to extract the features. Previous experiments proved that the convolution of 1 × 1 size had limited performance improvement for the model, so we only used it to adjust the number of channels and eliminate the aliasing of different features fusion.

This part of the pseudocode is shown in Algorithm 2. *i* indicates the serial number of the camera. *C2*, *C3*, *C4*, and *C5* indicate the four bottlenecks of ResNet. $x_{ic}$ and $F\left(x_{i},W_{i}\right)$ denote the feature map and result of the $c_{th}$ bottleneck in the $i_{th}$ camera. We merge the feature map of the last bottleneck through a 3 × 3 convolution layer and a 1 × 1 convolution layer and the feature map of the current bottleneck, then concatenate the merged result of each layer as the final outputs.

**Figure 2:**
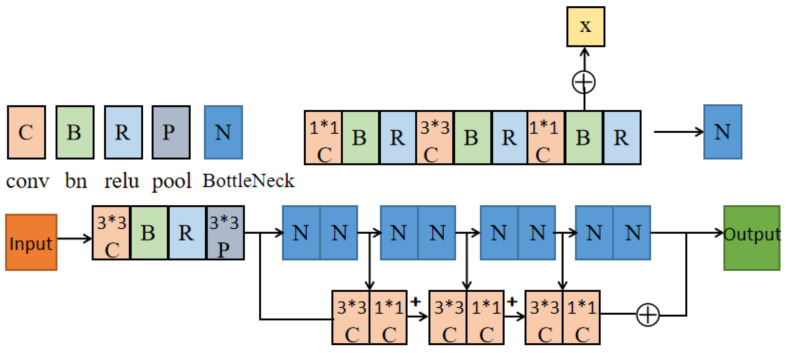
Double-branch convolution fusion structure.

As shown in Figure 2, the double-branch structure is used in this model, and the data is passed into the corresponding branches. We take the ResNet network as the first branch. However, some low-level semantic information also needs to be preserved. The second branch of the model consists of a 3 × 3 convolution layer and a 1 × 1 convolution layer, which are merged after adaptive averaging pooling and the RELU layer. Because ResNet has four bottlenecks, each bottleneck catches different information. The fusion layer includes the present bottleneck, a 3 × 3 convolution layer, a 1 × 1 convolution layer, and the last bottleneck. We then concatenate the result with each fusion layer.
**Algorithm 2** Double branch feature fusion process**Input:** 
The data of $image_{1}\ldots image_{N}$**Output:** 
A point that represents the center of the target1:initial i=0;2:$F\left(x_{i},W_{i}\right)$ is the residual part, usually consisting of two or three convolution operations;3:$W_{i}^{\prime }$ represents the convolution of 1*1;4:$W_{i}^{\prime \prime }$ represents the convolution of 3*3;5:**repeat**6:    **for** *C* in [C2,C3,C4,C5] **do**7:        $x_{ic}=x_{i(c-1)}+F\left(x_{i(c-1)},W_{i(c-1)}\right)$8:        $x_{i}^{\prime }=W_{i}^{\prime \prime }x_{i}$;9:        $x_{i}^{\prime }=W_{i}^{\prime }x_{i}^{\prime }$;10:        add $x_{i}^{\prime }$ to ans;11:    **end for**;12:    **for** *C* in [C2,C3,C4,C5] **do**13:        $res=res.concat(ans_{c})$;14:    **end for**;15:    res=res.concat(x);16:    $res=W_{i}^{\prime }res$;17:**until** (i>=N)

The structure of the double-branch can better extract features and make the model achieve better performance. Because the front layer often carries more data information, the model adds a fusion structure and then makes it fuse with the feature map before the last layer of the network. Because the output feature map of the branch structure has different channel numbers from the feature map obtained at the last layer, we adjust the channel numbers by an additional 1 × 1 convolution. Dilated convolution is adopted here. Dilated convolution can increase the sensing field of the convolution kernel while keeping the number of parameters unchanged, which also improves the performance of the model.

The structure of the double-branch can better extract features and make the model have better performance. Then, the global information is embedded by simply using the channel statistics generated $s\in R^{C}$ by the global average pool. Specifically, the cth element of s is computed by contracting the feature by the spatial dimension H×W with the following equation.

(1) $$S_{c}=\frac{1}{H\times W}\sum_{i=1}^{H}\sum_{j=1}^{W}x_{c}\left(i,j\right)$$

After that, they are added together with the following equation.

(2) $$F=\left(d_{\left[b,c,h,w\right]}+m_{\left[b,c,h,w\right]}\right)/2$$

where *D* is the feature graph output from branch 1, and *m* is the feature graph output from branch 2, and *b*, *c*, *h*, and *w* represent their batch, channel, height, and weight. The high-level semantic information is usually saved after ResNet downsampling, but the model cannot learn perfectly if low-order features are not saved. To save as much data information, the double-branch structure proposed by us loses relatively less data information. The model adds a 3 × 3 convolution layer behind each ResNet layer to extract features and store them, ensuring that the whole network can retain as much original information and maximize the role of data information. Because the number of channels of the output feature graph is different from that of the feature graph obtained at the last layer of the model, the feature graph output by the double-branch structure needs to go through an additional 1 × 1 convolution adjustment channel. The module can be naturally extended to aggregate multi-scale features without the help of FPN.

### 3.3. Center Points

The midpoint of the target is taken as the keypoint. In the Heatmap, the point is taken as the center of the circle, and the radius is *R* (*R* = 4). The value is filled with the Gaussian kernel function. Because there is only one category to be detected and the model converges quickly, no more iterations are needed to optimize the model. There is no need to add additional penalty terms to balance difficult samples and positive and negative examples. The keypoint detection process is shown in Algorithm 3. *p* denotes the center point of the target. $Y_{xyc}$ indicates the Gaussian function.
**Algorithm 3** Key point detection process**Input:** 
The data of $image_{1}\ldots image_{N}$**Output:** 
Gaussian heatmap with predicted points based on the original image1:initial i=0;2:$W_{i}^{\prime }$ represents the convolution of 1*1;3:x1,y1,x2,y2 corresponds to the left, up, right, down of the target;4:*r* be the downsampling parameter 45:Filling calculated value with Gaussian function $Y_{xyc}$6:**repeat**7:    **for** j∈len(objects) **do**8:        $p=\left(\frac{x_{1}+x_{2}}{2},\frac{y_{1}+y_{2}}{2}\right)$;9:        Mark image $Y\in {\left[0,1\right]}^{\frac{W}{R}\times \frac{H}{R}\times C}$;10:        $Y_{xyc}=\exp\left(-\frac{{\left(x-{\tilde{p}}_{x}\right)}^{2}+{\left(y-{\tilde{p}}_{y}\right)}^{2}}{2\sigma_{p}^{2}}\right)$;11:    **end for**;12:    $Y=W_{i}^{\prime }Y$;13:**until** (i>=N)

**Figure 3:**
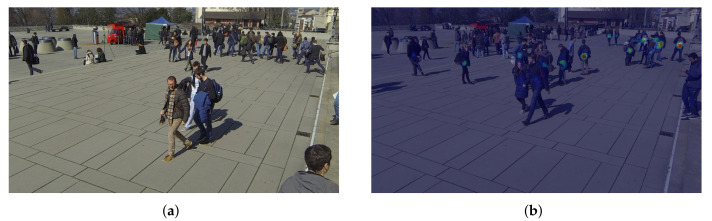
Central heat map results for pedestrian detection. (**a**) RGB image; (**b**) predict object center heatmap.

Because the inaccurate anchor box affects the feature mapping projection method, ground plane feature maps constructed via anchor-free feature perspective transformation are more accurate. In this paper, the target is presented through the center point, and some attributes of the target are regressed at the center point. The target detection problem becomes a standard critical point estimation problem. We pass the image into the fully convolutional network to obtain a thermal map. The peak point of the thermal map is the center point. The location of the peak point of each feature map predicts the width and height information of the target. Each target has only one positive anchor, and no NMS or grouping is used. The center points always contain more global information than other points, the position is easier for the model to fit. Therefore, the center point is adopted as the ground truth. The prediction results of the model are shown in Figure 3. The left is the RGB image, and the right is the results obtained by the model training.

Transforming a three-dimensional object or object in a spatial coordinate system into a two-dimensional image is called projection transformation. The size of the perspective projection of a three-dimensional object is inversely proportional to the distance from the body to the viewpoint (projection center). Compared with parallel projection, the perspective projection has a greater sense of depth and looks more realistic. We only need to know the specific position of the target in the top view, and the loss can be minimized by using the external parameters of the camera. The model extracts the features by convolution and then uses the feature map to perform the perspective transformation operation. The conversion between the 3D position (x,y,z) and the 2D image pixel coordinate (u,v) is done by a point-by-point transformation.

(3) $$s\left(\begin{array}{c} v\\ u\\ 1 \end{array}\right)=P_{\theta ,1}\left(\begin{array}{c} x\\ y\\ 1 \end{array}\right)$$

(4) $$P_{\theta ,1}\left(\begin{array}{c} x\\ y\\ 1 \end{array}\right)=\left[\begin{array}{cccc} \theta_{11} & \theta_{12} & \theta_{13} & \theta_{13}\\ \theta_{21} & \theta_{21} & \theta_{21} & \theta_{24}\\ \theta_{31} & \theta_{32} & \theta_{33} & \theta_{34} \end{array}\right]\left(\begin{array}{c} x\\ y\\ 1 \end{array}\right)$$

where *s* is a real scale factor, $P_{\theta ,1}$ is a 3 × 4 angle transformation matrix. [R|t] is a 3 × 4 joint rotation-translation matrix, which is the matrix of the external parameters, where *R* denotes the rotation and *t* denotes the translation.

The formula for obtaining the midpoint is as follows.

(5) $$p_{k}=\left(\frac{x_{1}^{k}+x_{2}^{k}}{2},\frac{y_{1}^{k}+y_{2}^{l}}{2}\right)$$

Our keypoints effectively use the internal features of objects and perceive the internal information of objects, reducing a large number of FP. The Anchor mechanism is completely abandoned, and the IOU between the preselection box and the real box is not needed to consume additional resources. There is no need to generate a series of position coordinates for coordinate regression and classification prediction, which reduces the processing time of generating anchor encoding and decoding. It helps the model to locate the target quickly and the model calculation is simple. We do not need to enlarge the resolution of the image, so the running time of the model can be reduced.

Model regression stage adopted the function of *L*_1_ smooth. The formula is shown below.

(6) $${\mathrm{smooth}}_{L_{1}}\left(x\right)=\left\{\begin{array}{cc} 0.5x^{2} & \;\mathrm{if}\;|x|<1\\ |x|-0.5 & \;\mathrm{otherwise}\; \end{array}\right.$$

**Figure 4:**
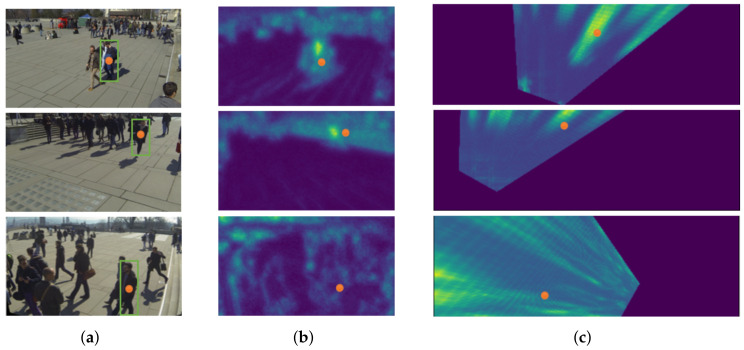
Feature projection process diagram. (**a**) Input: N RGB views; (**b**) feature maps; (**c**) projected feature maps.

As in Figure 4, center points in the bounding box are used as the ground truth, and the center feature map is obtained after feature extraction. The orange dots represent a specific ground plane location and its corresponding pixel in different views. The green bounding boxes refer to anchor boxes whose center (human center point) is at that ground plane location.

## 4. Experiments

For comparison purposes, we downsample the 1080 × 1920 RGB images to $H_{i}=720,$$W_{i}=1280$ and remove the last two layers (global average pooling; classification output) in ResNet-18 like MVDet. We use dilated convolution to replace the normal convolution. This results in a 8 × downsample from the 720 × 1280 input. The double-branch structure uses the 3 × 3 convolution to extract the feature and uses 1 × 1 convolution to change channel numbers. We use the center point to indicate the target to calculate. We use an SGD optimizer with a momentum of 0.5, L2-normalization of 5 × $10^{-4}$. We use the one-cycle learning rate scheduler with the max learning rate set to 0.1, and train for 30 epochs with the batch size set to 1. We finish all the experiments on one RTX-2080ti GPU and one RTX Titan GPU.

### 4.1. Datasets

The Wildtrack dataset uses joint visual information from multiple simultaneous cameras to improve detection performance. It was captured by seven static cameras in a public open area, where a large number of standing and walking pedestrians were gathered. Together with the camera frames, this dataset provides an accurate joint (extrinsic and intrinsic) calibration, as well as 400 annotated frames from 7 series, detected at a rate of 2 frames per second. This produced over 40,000 bounding frames, delineating each person present in the field of interest, with over 300 people in total. On average, there were 20 people on each edge Wildtrack dataset, and each location in the scene was covered by 3.74 cameras.

The MultiviewX dataset is a new synthetic dataset for multi-view pedestrian detection. The MultiviewX dataset covers a slightly smaller area of 16 meters by 25 m. The dataset quantifies the ground plane like a 640 × 1000 grid. There are six cameras in the MultiviewX dataset with overlapping fields of view, each outputting images at 1080 × 1920 resolution. Four hundred frames are annotated in MultiviewX at two frames/second (the same as Wildtrack). On average, there were 4.41 cameras at the exact location, with approximately 40 people per frame, twice as many as in the Wildtrack dataset. A comparison of the two datasets is shown in Table 1.

Evaluation metrics: Following [24]. We used 90% of the dataset for training and the remaining 10% for testing, using Precision, Recall, MODA, and MODP [25] as the evaluation metrics. MODP (Multiple Object Detection Precision) evaluates the localization accuracy, while MODA (Multiple Object Detection Accuracy) can account for false positives and false negatives. Because MODA takes into account both false positives and false negatives, MODA is the main evaluation metric.

As described before, $G_{i}^{\left(t\right)}$ denotes the $i^{th}$ ground truth object in frame *t*. $D_{i}^{\left(t\right)}$ denotes the detected object for $G_{i}^{\left(t\right)}$.

(7) $$OverlapRatio=\sum_{i=1}^{N_{\mathrm{mapped}\;}^{t}}\frac{\left|G_{i}^{\left(t\right)}\cap D_{i}^{\left(t\right)}\right|}{\left|G_{i}^{\left(t\right)}\cup D_{i}^{\left(t\right)}\right|}$$

We can calculate the multiple object detection precision for each frame *t*. The mapped $N_{t}$ is the number of mapped sets of objects in the coordinate system *t*:

(8) $$\mathrm{MODP}\left(t\right)=\frac{\left(\;\mathrm{Overlap}\;\mathrm{Ratio}\;\right)}{N_{\mathrm{mapped}\;}^{t}}$$

Consider that in each frame *t*, $m_{t}$ indicates a missing number, $f_{pt}$ indicates the number of false alarms, $c_{m}$ and $c_{f}$ is a function of the cost of missed and false alarm penalties, $N_{t}^{G}$ is the number of Ground Truths in frame *t*. We can calculate MODA:

(9) $$\mathrm{MODA}\left(t\right)=1-\frac{c_{m}\left(m_{t}\right)+c_{f}\left(f_{Pt}\right)}{N_{t}^{G}}$$

### 4.2. Comparison with Different Methods

As shown in Table 2, we can compare multiview aggregation and the backbone in different methods.

The new DCNN consists of five convolutional layers, two max-pooling layers, three fully-connected layers, and a final 1000-dimensional output. The last two layers are discarded and replaced by random initializations.

We compare the performance of our model with different multiview pedestrian detection methods. It can be seen that the performance of the proposed method has been steadily improved in both Wildtrack and MultiviewX datasets. The single-target Precision and Recall rates usually appear very high in these models. Therefore, MODA and MODP are taken as the primary evaluation metrics, and the final results are shown in Table 3. On the Wildtrack dataset, our model achieves 90.0% MODA, a +1.8% increase over previous state-of-the-art models. On the MultiviewX dataset, MVDet achieves 89.5% MODA, a +5.6% increase over our implementation of MVDet. Our model also achieves the highest MODP and recall on both datasets, but falls slightly behind MVDet interms of precision on the Wildtrack dataset.

Spatial aggregation with CRF and mean field inference brings forward increases of +6.3% and +5.2% on the two datasets, going from Deep MCD to Deep-Occlusion. Large kernel convolutions brings forward a +14.1% MODA increase on Wildtrack dataset, and a +8.7% performance increase on the MultiviewX dataset, going from Deep-Occlusion to MVDet. The double-branch structure and center points detection brings forward a +1.8% MODA increase on the Wildtrack dataset, and a +5.6% performance increase on the MultiviewX dataset, going from MVDet to our model.

It can be seen from Table 3 and Table 4 that only the precision of the model does not improve on the Wildtrack dataset, while other evaluation matrices all reach the highest on different datasets. Since MultiviewX is a simulation dataset and the features are easy to capture, the accuracy is improved to a more noticeable level. All evaluation matrics on this dataset have reached the highest level at present.

### 4.3. Ablation Experiments

A double-branch multi-layer convolution integration module is proposed in this paper. We use the expansion convolution to expand the receptive field, which supports exponential expansion of the receiving field without reducing the resolution. The method extracts and utilizes the features based on double-branch multi-layer convolution integration module and predicts based on the keypoint. We conducted a comparative test on the two parts, and the results are shown in Table 5 and Table 6.

As seen from Table 5 and Table 6, our method of using center points instead of positive sampling points is more competitive than MVDet, which used a head-foot point for prediction. Our method maximized the use of global information and only used one point to indicate each target, which greatly improves the model speed. Even though the proposed keypoints detection method does not have a significant improvement effect, we could further extract features based on the double-branch features fusion to make the model show better results in MODA, MODP, and Recall. Because the keypoints are more convenient for various calculations for the model, they show better performance when combined with the double-branch multi-layer convolution fusion module.

**Figure 5:**
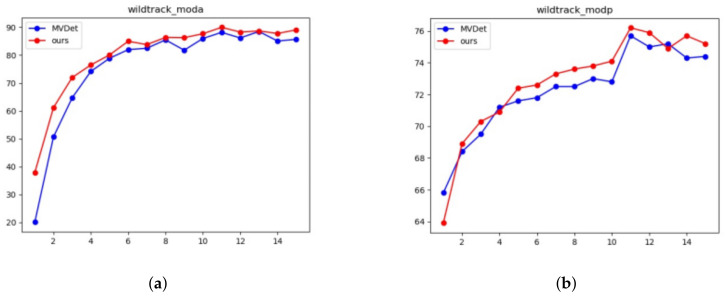
Comparison with state-of-the-art model in Wildtrack datasets. (**a**) Wildtrack MODA; (**b**) Wildtrack MODP.

**Figure 6:**
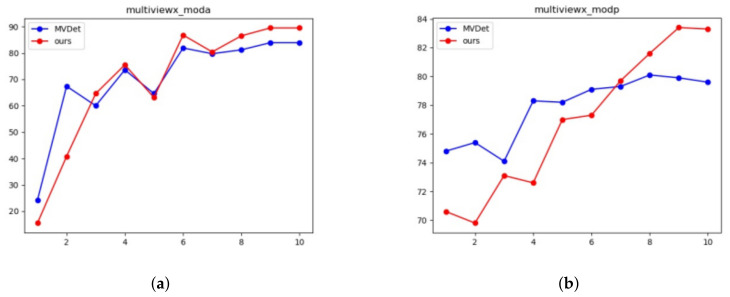
Comparison with state-of-the-art model in MultiviewX datasets. (**a**) MultiviewX MODA; (**b**) MultiviewX MODP.

The changing trend of MODA and MODP of the proposed model and the current state-of-the-art model is compared in Figure 5 and Figure 6. It can be seen from the results that our model always performs better in Wildtrack and MultiviewX datasets.

**Figure 7:**
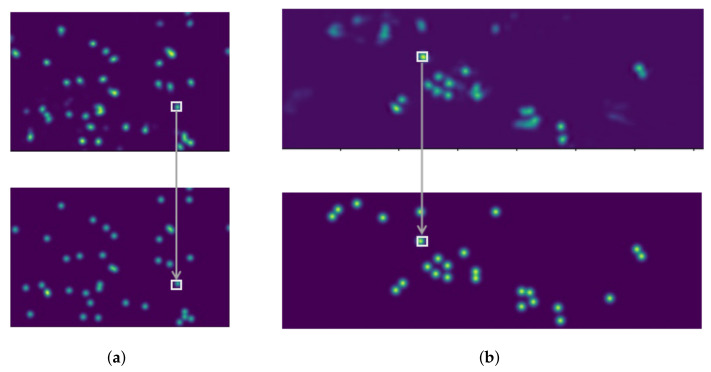
Prediction and results under different datasets. (**a**) MultiviewX; (**b**) Wildtrack.

Figure 7 shows the final results of our model. The bottom part of Figure 7 is the Ground Truth corresponding to a scene in the two datasets, and the small box represents the Ground Truth corresponding to the model detection results. Each point represents the position of a target in the corresponding top view. It can be seen that the results obtained by the model are consistent with the label, and there are almost no missed pedestrian targets.

### 4.4. Contribution of Speed

Real-time is very important in the multi-perspective pedestrian detection task. Keypoints detection completely abandons the anchor mechanism, which has no need for consuming extra resources to calculate the IOU. It generates a series of position coordinates for coordinate regression and classification prediction and reduces the time to generate anchor and conduct encoding and decoding processing. There is no time-consuming post-processing process for the center point to help the model locate the target position quickly. The model calculation steps are simple, and we do not need to enlarge the resolution of the image when processing. The detection method of keypoints and double-branch feature fusion module can achieve each other, presenting the effect of 1 + 1 > 2. Each dataset has *N* cameras, the images from *N* angles are treated as a group, The FPS indicate several groups per second. The comparison of running times on Wildtrack and MultiviewX are shown in Table 7.

The double-branch structure takes a certain amount of time due to feature extraction. However, the detection method of key points can make the model run faster. It can be seen from Table 5 that the speed of our model in the Wildtrack and MultiviewX datasets is accelerated to varying degrees.

## 5. Conclusions

In this paper, our base detection model introduces a double-branch feature fusion structure based on the lightweight network to further refine the extraction of features. We use center points to replace the positive sampling points and make the model more flexible. The idea reduces the number of parameters, speeding up the model, and avoids the negative impact of the anchor frame mechanism on the model. The keypoints detection is adapted to the double-branch feature fusion model. A multi-view aggregation approach with feature projection allows the generated bird’s eye view to aggregate multi-camera pedestrian information. Our model achieves MODA of 90.0% on the Wildtrack dataset, which is higher than the previous state-of-the-art by 1.8%. Very competitive results are also performed on the MultiviewX simulation dataset, which is higher than the previous state-of-the-art by 5.6%. 

## Figures and Tables

**Table 1:** Comparison of Wildtrack and MultiviewX.

Dataset	Camera Number	Resolution	Area	Crowdedness
Wildtrack	7	1080×1920	12×36 m${}^{2}$	20 person/frame
MultiviewX	6	1080×1920	16×25 m${}^{2}$	40 person/frame

**Table 2:** Multiview aggregation and backbone in different methods.

Method	Multiview Aggregation	Backbone
RCNN andd clustering	detection results	The new DCNN
DeepMCD	anchor box features	GoogLeNet
Deep-Occlusion	anchor box features	VGG
MVDet	feature maps	ResNet-18
Ours	feature maps	ResNet-18+feature fusion

**Table 3:** Performance comparison with different methods for the Wildtrack dataset.

Method	MODA/%	MODP/%	Precision/%	Recall/%
RCNN and clustering	11.3	18.4	68	43
DeepMCD	67.8	64.2	85	82
Deep-Occlusion	74.1	53.8	95	80
MVDet	88.2	75.7	94.7	93.6
Ours	90.0	76.2	94.5	94.7

**Table 4:** Performance comparison with different methods for the MultiviewX dataset.

Method	MODA/%	MODP/%	Precision/%	Recall/%
RCNN and clustering	18.7	46.4	63.5	43.9
DeepMCD	70.0	73.0	85.7	83.3
Deep-Occlusion	75.2	54.7	97.8	80.2
MVDet	83.9	79.6	89.5	85.9
Ours	89.5	83.4	98.1	91.3

**Table 5:** Results of the ablation experiment for the Wildtrack dataset.

Method	MODA/%	MODP/%	Precision/%	Recall/%
MVDet	88.2	75.7	94.7	93.6
Keypoints	88.7	75.3	95.2	94.5
Feature fusion	88.2	75.3	95.8	94.1
Ours	90.0	76.2	94.5	94.1

**Table 6:** Results of the ablation experiment for the MultiviewX dataset.

Method	MODA/%	MODP/%	Precision/%	Recall/%
MVDet	83.9	79.6	89.5	85.9
Keypoints	84.7	80.7	97.8	86.6
Feature fusion	88.8	82.7	98.6	90.1
Ours	89.5	83.4	98.1	91.3

**Table 7:** Running time of the models on different test sets.

FPS	Wildtrack/*s*	MultiviewX/*s*
MVDet	3.42	4.09
Ours	3.58	4.30

## Data Availability

Data is contained with in the article.
